# Pasireotide for Refractory Hypoglycemia in Malignant Insulinoma- Case Report and Review of the Literature

**DOI:** 10.3389/fendo.2022.860614

**Published:** 2022-04-19

**Authors:** Sandrine Oziel-Taieb, Jemima Maniry-Quellier, Brice Chanez, Flora Poizat, Jacques Ewald, Patricia Niccoli

**Affiliations:** ^1^ Department of Medical Oncology, Institut Paoli-Calmettes, European Neuroendocrine Tumor Society (ENETS) Center of Excellence, Institut Paoli-Calmettes Neuroendocrine Tumor (IPC NET) Center, Marseille, France; ^2^ Department of Biopathology, Institut Paoli-Calmettes, European Neuroendocrine Tumor Society (ENETS) Center of Excellence, Institut Paoli-Calmettes Neuroendocrine Tumor (IPC NET) Center, Marseille, France; ^3^ Department of Surgical Oncology, Institut Paoli-Calmettes, European Neuroendocrine Tumor Society (ENETS) Center of Excellence, Institut Paoli-Calmettes Neuroendocrine Tumor (IPC NET) Center, Marseille, France

**Keywords:** pasireotide, refractory hypoglycemia, neuroendocrine tumor, pancreatic neuroendocrine cancer, malignant insulinoma

## Abstract

Malignant insulinomas are functional neuroendocrine tumors of the pancreas and the primary cause of tumor-related hypoglycemia. Malignant insulinoma is rare and has a poor prognosis. We report a case of metastatic malignant insulinoma in a 64-year-old female patient with severe and refractory hypoglycemia. After several ineffective locoregional and systemic therapeutic lines for the secretory disease, the introduction of pasireotide, a second-generation somatostatin analog, provided an improved clinical and secretory evolution both quickly and sustainably, with an excellent safety profile. Pasireotide is an effective and well-tolerated therapy in the treatment of refractory hypoglycemia in metastatic insulinoma.

## Introduction

Insulinomas, functional neuroendocrine tumors of the pancreas, are the leading tumor-related cause of hypoglycemia with an estimated incidence of 1-3 per million per year (1) and 4% to 14% of insulinomas are characterized as malignant due to the presence of locoregional extension and/or metastatic spread. Metastatic insulinomas are almost well differentiated and the presence of liver metastases worsens their prognosis (2). Despite a large therapeutic arsenal including somatostatin analogs, debulking surgery, hepatic arterial embolization, percutaneous local tumor ablation, targeted therapies, Peptide Receptor Radionuclide Therapy (PRRT), and chemotherapy, malignant insulinoma still has a poor prognosis (3). Glycemic control and tumor volume control are the two therapeutic objectives.

We report a case of metastatic malignant insulinoma in a 64-year-old female patient with severe and refractory hypoglycemia despite having received several lines of treatment. The introduction of pasireotide, a second-generation somatostatin analog, has resulted in rapid and lasting control of blood glucose levels and a clinical benefit.

## Case Description

A 64-year-old woman with no significant past medical history presented, in February 2016, with recurrent episodes of non-fasting hypoglycemia with neuroglycopenic symptoms, which resolved with meal. She was in good general condition with a performance status of 0, a weight of 62 kg, and a height of 171 cm.

Thoracoabdominopelvic computed tomography (CT) and magnetic resonance imaging (MRI) of the liver revealed a hypervascular mass in the tail of the pancreas associated with liver bilobar metastatic spread. An endoscopic ultrasound found a 16 x 30 mm mass in the tail of the pancreas, demonstrated a paucicellular synaptophysin positive sample with a Ki-67 of less than 5%. A liver biopsy of a metastasis confirmed a well-differentiated grade 2 neuroendocrine tumor with Ki-67: 4%. In initial laboratory results, elevated Chromogranin A (CgA) levels of 2219 ng/mL (27-94), normal NSE levels of 12.5 ng/mL (<17), normal C peptide levels of 0.38 pmol/L (0.3-1.4) and normal insulin levels of 96 pmol/L (18-173) and glucose level of 0.30g/L (0.74- 1.06) were detected.

We initiated a somatostatin analog treatment with standard dose long acting-release (LAR) octreotide (30 mg). From the first injection of the drug an exacerbation of hypoglycemic crises occurred motivating the early discontinuation of octreotide after a unique dose. In July 2016, the patient was hospitalized for hypoglycemic coma. Diazoxide administration and trans-arterial chemoembolization (TACE) of the right hepatic lobe were performed, improving glycemic control with a significant reduction of hypoglycemic episodes.

In March 2017, attempted reintroduction, for tumor control, of another LAR somatostatin analog, lanreotide (120 mg every 4 weeks), rapidly lead to a recurrence of hypoglycemic episodes and resulted in definitive discontinuation of first-generation somatostatin analogs in June 2017. In August 2017, everolimus was introduced for glycemic control but had to be discontinued early, ten days later, due to grade 3 thrombocytopenia.

In September 2017, a second TACE was performed and resulted in blood glucose levels control. A third TACE in April 2018 was indicated for hepatic progressive disease. Hypoglycemic events quickly recurred in July 2018 requiring 30% glucose infusion.

**Figure 1:**
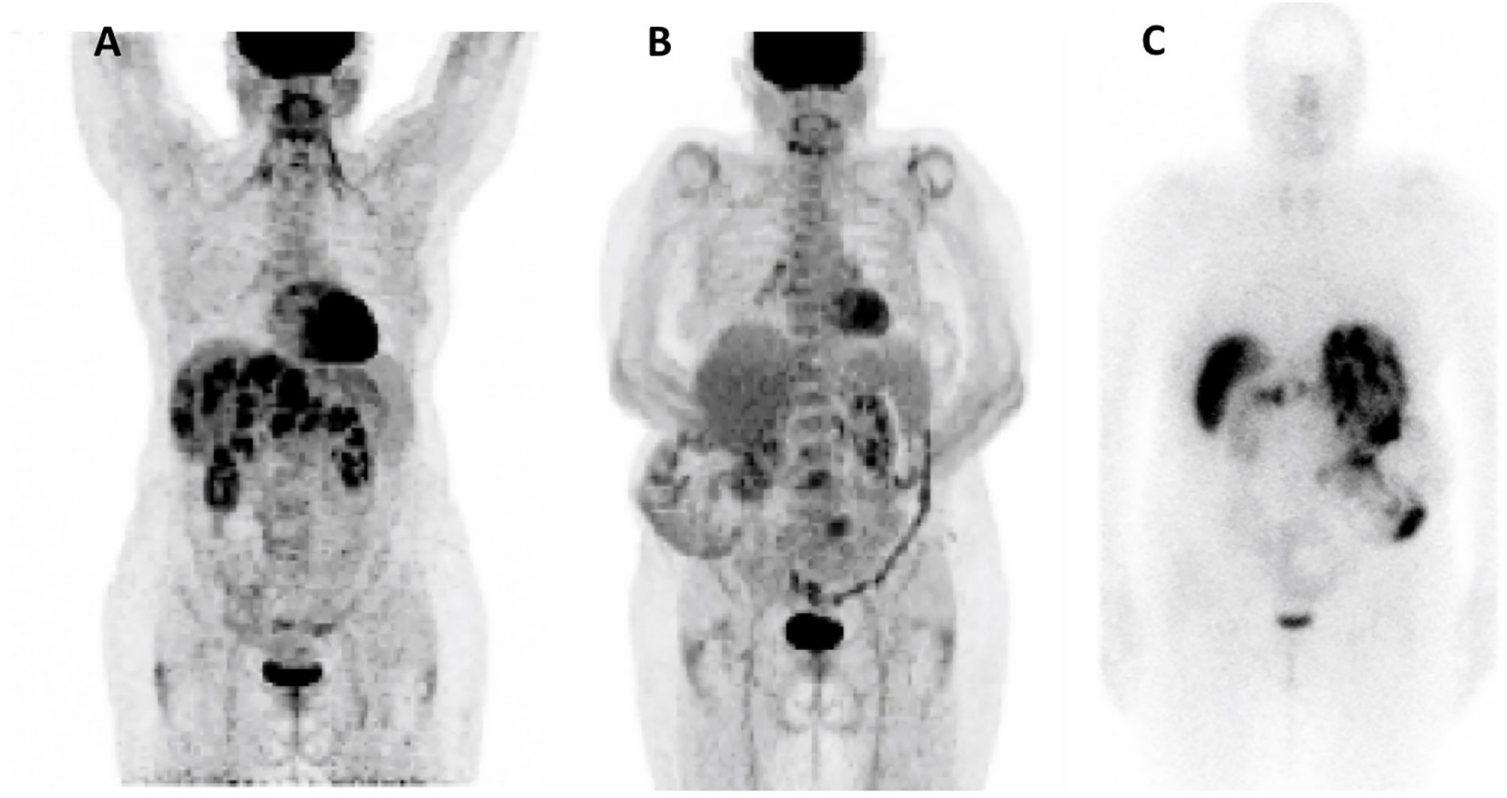
Nuclear Imaging. **(A)** ^18^F-FDG-PET/CT Oct 2018; **(B)** ^18^F-FDG-PET/CT Dec 2020; **(C)** Octreoscan Dec 2020.

In October 2018, ^18^F-FDG positron emission tomography/computed tomography (^18^F-FDG-PET/CT) revealed multiple hypermetabolic lesions of the liver (SUV_max_=18.3), pancreas (SUV_max_= 7.3), and lymph nodes (SUV_max_ =7.8) (**Figure 1** **).** From December 2018 to October 2019, everolimus was reintroduced at a reduced dose of 5 mg, due to the previous hematological toxicity, allowing radiological stable disease without control of hypoglycemia events.

**Figure 2:**
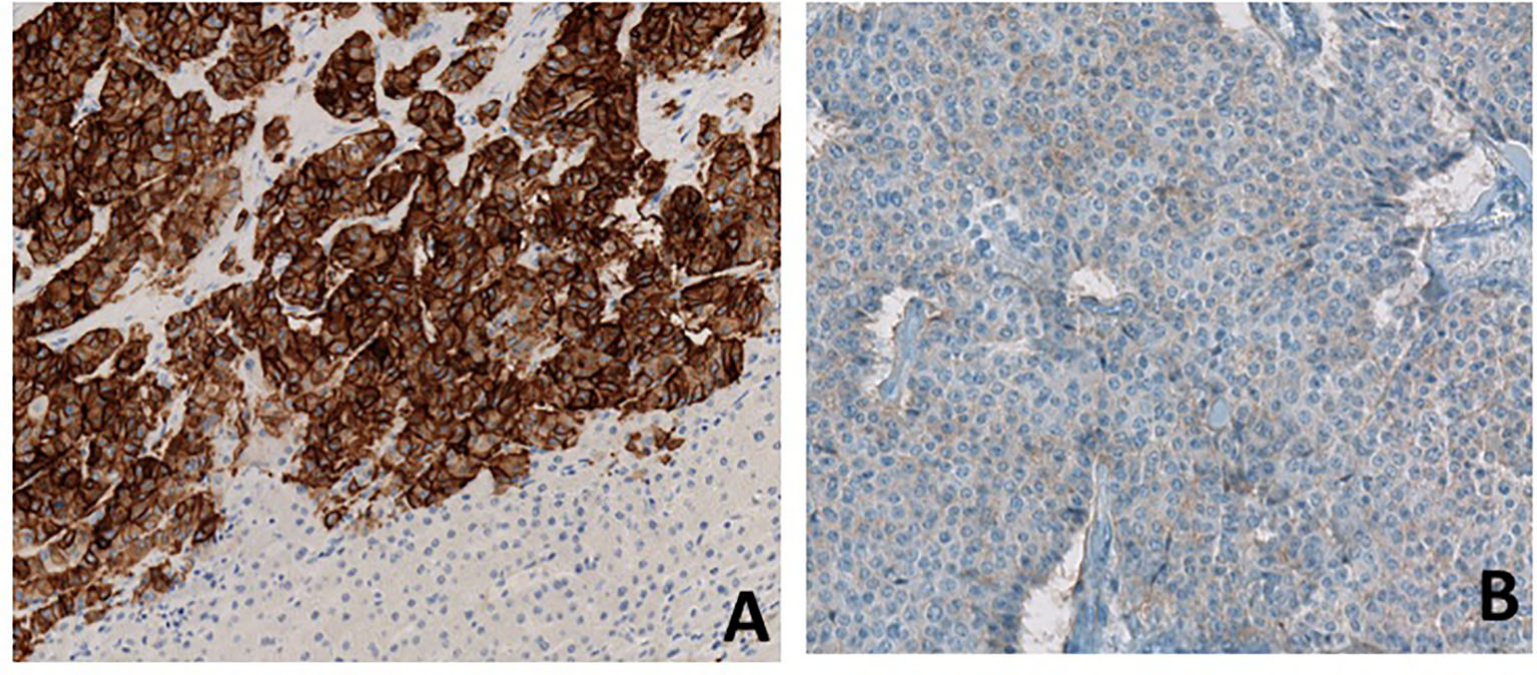
Immunostaining for SSTR2 **(A)** and SSTR5 **(B)**. Immunostaining of metastatic hepatic lesion shows high expression of SSTR2 and low expression of SSTR5.

After a fourth ineffective TACE, a hepatic debulking surgery was performed in June 2019. Hepatic histological analysis confirmed a well-differentiated grade 2 neuroendocrine tumor with an increased Ki-67: 18% (versus 4% in 2016). Immunostaining showed strong somatostatin receptor 2 (SSTR2) positivity and a weak SSTR5 expression (**Figure 2**)

**Figure 3:**
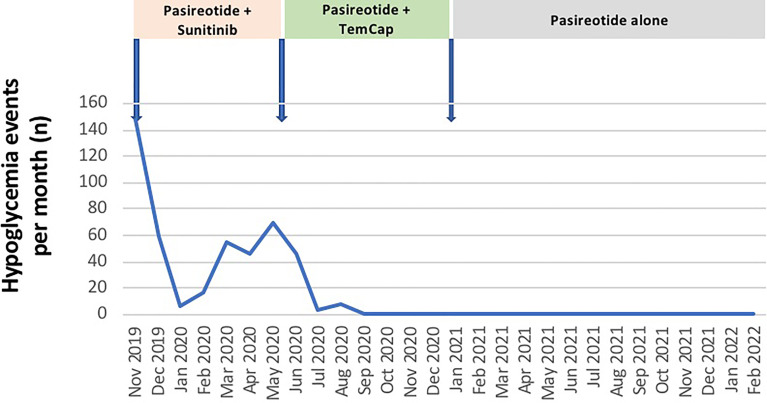
Hypoglycemia events per month since beginning of Pasireotide.

The patient was hospitalized from the end of October 2019 to the end of December 2019 for severe refractory hypoglycemia, seizures and a deterioration of the clinical status. After everolimus discontinuation, sunitinib was started at a dose of 37.5 mg/day. Despite continuous 30% glucose infusions and corticosteroid therapy, glycemic control was insufficient. Subcutaneous short-acting pasireotide at a dose of 0.9 mg every 12 hours was initiated, resulting in rapid clinical status improvement and a prompt decrease of the frequency and severity of hypoglycemic events counted by a continuous glucose monitoring (**Figure 3**).

**Figure 4:**
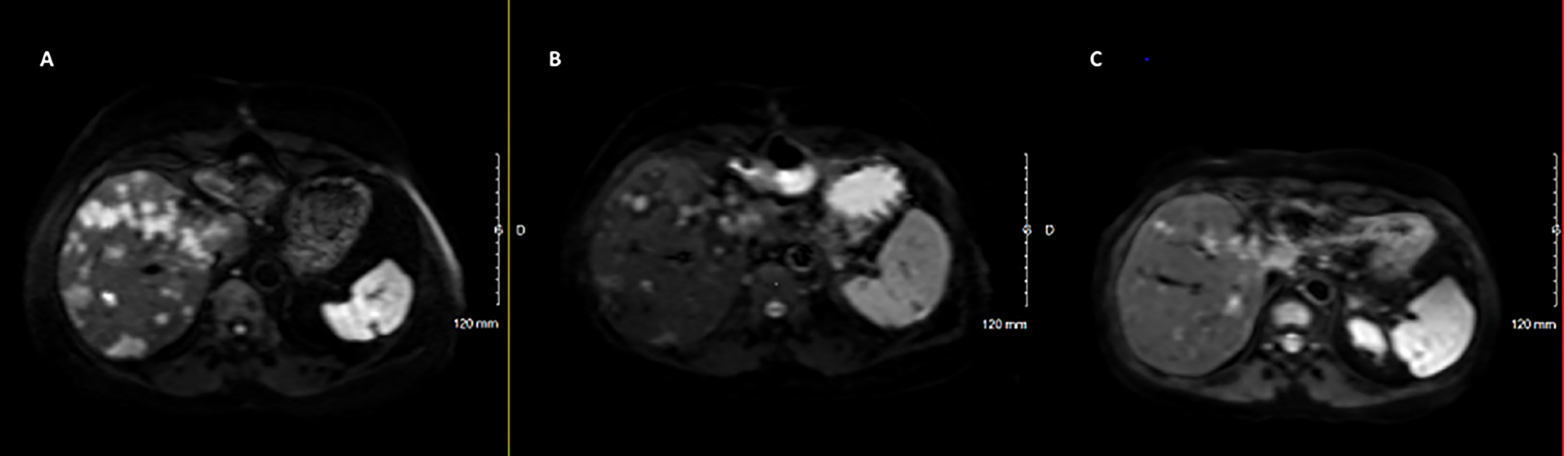
Radiological (MRI Diffusion) to CAPTEM and pasireotide. **(A)** June 2020, before CAPTEM; **(B)** December 2020, after 6 cycles of CAPTEM; **(C)** February 2022, pasireotide alone.

**Figure 5:**
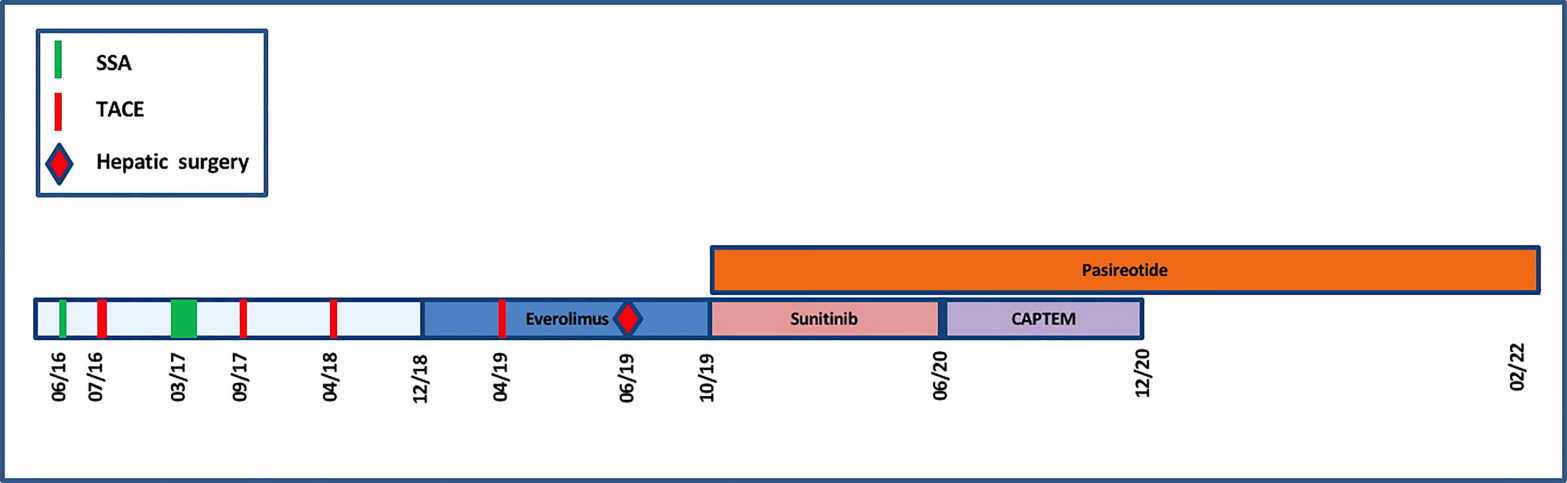
Timeline representing the anti-proliferative and anti-secretory treatments since diagnosis.

Sunitinib was discontinued in June 2020 due to both recurrent hypoglycemia and an increase in CgA levels to 55173ng/ml with radiological stable disease. A capecitabine and temozolomide combination (CAPTEM) was administered while continuing pasireotide. Then, the patient was able to be managed at home with good glycemic control. Three months after starting the chemotherapy in association with pasireotide, a good tumor and secretory response was observed allowing progressive discontinuation of corticosteroids and glucose infusions. CAPTEM was continued until December 13, 2020 with dose reduction due to grade 3 thrombocytopenia. Short-acting pasireotide has been replaced by 60 mg LAR pasireotide in December 2020 to ensure better comfort. At the same time, a ^18^F-FDG-PET/CT revealed a complete metabolic response compared to 2018 and Octreoscan showed intense hyperfixation of all secondary hepatic lesions in favor of SSTR2 expression (**Figure 1** **).** Tumoral response was assessed by hepatic MRI showing a partial response on December 2020 sustained through February 2022 (**Figure 4**). CAPTEM was discontinued after 6 cycles due to a good partial response and persistent grade 2 trombocytopenia. The anti-proliferative and anti-secretory treatments since diagnosis are detailed in **Figure 5**.

Pasireotide has been continued without interruption since its introduction. The patient has been treated as an outpatient for 26 months with monthly intramuscular injections of pasireotide LAR. Given the sustained glycemic control, the dose of pasireotide LAR could be progressively decreased from 60 mg to a dose of 20 mg in February 2022. Blood glucose levels have been completely normalized for over 18 months at this point, resulting in a significant improvement in quality of life.

## Discussion and Conclusion

We report here a case of metastatic insulinoma with severe and refractory hypoglycemia that showed a good and durable secretory response to pasireotide, as well as good tumor response to the combination of CAPTEM and pasireotide.

Pasireotide is a second-generation multi-somatostatin receptor ligand with an affinity for four of the five SSTRs especially SSTR5, followed by SSTR2, SSTR3, and SSTR1. It is currently approved for the treatment of Cushing’s disease (4) and acromegaly, with a safety profile similar to the first-generation somatostatin analogs, octreotide or lanreotide, but with an increased risk of hyperglycemia (5). In patients treated with pasireotide for Cushing’s disease or acromegaly, the hyperglycemic effect may be explained by the different binding affinities to the different SSTR subtypes and by suppression of insulin secretion from normal pancreatic islet *via* SSTR5 activation.

Only a few cases of metastatic insulinoma treated with pasireotide have been described in the literature. Tirosh et al. described a case of metastatic insulinoma treated with pasireotide provided good glycemic control, but not antitumor efficacy, when compared to lanreotide and everolimus (6). Siddiqui et al. also reported a case of metastatic insulinoma presenting refractory hypoglycemia despite diazoxide and octreotide treatment with rapid control *via* pasireotide, which was finally stopped due to diabetes (7). Finally, Sileo et al. reported a case of benign insulinoma in a patient who was a poor candidate for surgery because of elderly and comorbidities and who achieved preoperative glycemic control with pasireotide, allowing for surgery of the pancreatic lesion in optimal clinical and biological conditions (8).

Unfortunately, pasireotide has not yet demonstrated antitumor efficacy in NETs, and it remains unknown whether pasireotide has greater antiproliferative effects than octreotide and lanreotide (9). The efficacy of combination therapy with pasireotide and everolimus in NETs is also controversial, with a reportedly higher response rate but without significant benefit in PFS compared to everolimus alone (10, 11). A phase II clinical trial (NCT01253161) assessed the clinical activity of pasireotide in treatment-naïve patients with metastatic NETs and showed that patients with low hepatic tumor burden, normal baseline chromogranin A, and high tumoral SSTR5 expression experienced the most favorable effect. SSTR 1-5 expression data was available for nearly all patients in this study (12). Thus, further study is required to determine the precise antiproliferative effect of pasireotide in NETs patients irrespective of SSTR expression. In our case, the patient strongly expressed SSTR2 on liver metastases but SSTR5 expression was low (**Figure 2**). However, the expression of SSTRs can be heterogeneous in neuroendocrine tumors and nothing can be concluded from the low positivity of SSTR5 on a single metastatic sample.

In this case, PRRT was not proposed initially due to the high hypermetabolism at ^18^F-FDG-PET/CT in 2018. However, metabolic imaging data from 2020 would be encouraging to indicate PRRT in case of future progression since the disease strongly expresses SSTR2 and no longer shows FDG hypermetabolism (**Figure 1**).

The patient exhibited hepatic stable disease with the combination of sunitinib and pasireotide. The antitumor efficacy of sunitinib in PNETs has been proven with a majority of stable disease (13).

In our case, CAPTEM provided a prolonged partial response of the metastatic disease in accordance with literature data showing high and durable response rate in metastatic neuroendocrine tumors (14). The partial response was maintained 14 months after stopping chemotherapy while the patient was only receiving pasireotide. Then antitumor efficacy of pasireotide cannot be specifically assessed in this case.

## Conclusion

Pasireotide provided rapid glycemic control in a patient with metastatic insulinoma who presented refractory hypoglycemia despite several prior lines of treatment. The combination with temozolomide-capecitabine resulted in liver tumor response with maintenance of excellent glycemic control. Pasireotide may be a therapeutic alternative in the treatment of metastatic insulinoma with refractory tumor induced-hypoglycemia.

## Data Availability Statement

The raw data supporting the conclusions of this article will be made available by the authors, without undue reservation.

## Ethics Statement

Ethical review and approval was not required for the study on human participants in accordance with the local legislation and institutional requirements. The patients/participants provided their written informed consent to participate in this study.

## Author Contributions

SO-T and JM-Q wrote the first draft of the manuscript. SO-T, JM-Q, BC, FP and JE made contributions to the acquisition of the clinical data. SO-T and PN made critical revisions and approval final version. All authors contributed to the article and approved the submitted version.

## Conflict of Interest

The authors declare that the research was conducted in the absence of any commercial or financial relationships that could be construed as a potential conflict of interest.

## Publisher’s Note

All claims expressed in this article are solely those of the authors and do not necessarily represent those of their affiliated organizations, or those of the publisher, the editors and the reviewers. Any product that may be evaluated in this article, or claim that may be made by its manufacturer, is not guaranteed or endorsed by the publisher.
